# Irritability in ADHD: Associations with depression liability

**DOI:** 10.1016/j.jad.2017.03.050

**Published:** 2017-06

**Authors:** Olga Eyre, Kate Langley, Argyris Stringaris, Ellen Leibenluft, Stephan Collishaw, Anita Thapar

**Affiliations:** a MRC Centre for Neuropsychiatric Genetics and Genomics, Division of Psychological Medicine and Clinical Neurosciences, Cardiff University, Wales, UK; b School of Psychology, Cardiff University, Wales, UK; c Institute of Psychiatry, Psychology and Neuroscience, Kings College London, London, UK; d Section on Bipolar Spectrum Disorders, Emotion and Development Branch, National Institute of Mental Health, Bethesda, MD, USA

**Keywords:** ADHD, DMDD, Irritability, Depression

## Abstract

**Background:**

Irritability and the new DSM-5 diagnostic category of Disruptive Mood Dysregulation Disorder (DMDD) have been conceptualised as related to mood disorder. Irritability is common in Attention Deficit Hyperactivity Disorder (ADHD) but little is known about its association with depression risk in this group. This study aims to establish levels of irritability and prevalence of DMDD in a clinical sample of children with ADHD, and examine their association with anxiety, depression and family history of depression.

**Methods:**

The sample consisted of 696 children (mean age 10.9 years) with a diagnosis of ADHD, recruited from UK child psychiatry and paediatric clinics. Parents completed the Child and Adolescent Psychiatric Assessment, a semi-structured diagnostic interview, about their child. This was used to establish prevalence of DMDD, anxiety disorder and depressive disorder, as well as obtain symptom scores for irritability, anxiety and depression. Questionnaires assessed current parental depression, and family history of depression.

**Result:**

Irritability was common, with 91% endorsing at least one irritable symptom. 3-month DMDD prevalence was 31%. Children with higher levels of irritability or DMDD were more likely to have comorbid symptoms of anxiety, depression and a family history of depression.

**Limitations:**

Results are based on a clinical sample, so may not be generalizable to children with ADHD in the general population.

**Conclusions:**

Irritability and DMDD were common, and were associated with markers of depression liability. Longitudinal studies are needed to examine the association between irritability and depression in youth with ADHD as they get older.

## Highlights

• Prevalence of DMDD was 31% in this clinical sample of children with ADHD.
• DMDD was associated with increased impairment and comorbidity.
• Irritability and DMDD were associated with markers of depression liability.

## 1 Introduction

Attention-Deficit/Hyperactivity Disorder (ADHD) is a common, impairing neurodevelopmental disorder that is associated with poor adult mental health outcomes (Klein et al., 2012). Depression is a common comorbidity (Spencer et al., 1999) that usually develops post-pubertally after ADHD onset. Young people with ADHD and depression are more impaired than those with ADHD or depression alone (Blackman et al., 2005). Therefore, identifying young people with ADHD who are at risk of depression is important, in terms of facilitating early intervention or prevention. Although depression risk factors have been identified in the general population, these may not generalise to young people with ADHD, especially as children with ADHD are predominantly male.

Irritability is common in ADHD, even though it is not a defining diagnostic feature. Irritability can be described as a propensity to react with anger, grouchiness, or tantrums disproportionate to the situation (Stringaris and Goodman, 2009a) and when included in the broader definition of emotional dysregulation, it is present in around 25–45% of children with ADHD (Shaw et al., 2013). In recent years an “irritable” dimension of Oppositional Defiant Disorder (ODD) has been identified (Stringaris and Goodman, 2009b). This includes the items “often loses temper”, “is often angry and resentful”, and “is often touchy or easily annoyed by others”, all of which are common in ADHD. This irritable dimension has been associated with elevated risk of emotional disorders and depression in the general population (Krieger et al., 2013, Stringaris and Goodman, 2009a, Stringaris et al., 2012, Vidal-Ribas et al., 2016, Whelan et al., 2013), but it is not known whether high levels of these symptoms in ADHD are also an early marker of mood problems.

More recently, severely impairing childhood chronic irritability has been conceptualised as a new diagnostic category in the mood disorders section of the Diagnostic and Statistical Manual of Mental Disorders, fifth edition (DSM-5) (American Psychiatric Association, 2013). This new diagnostic category, known as Disruptive Mood Dysregulation Disorder (DMDD), is characterised by severe temper outbursts that are grossly out of proportion in intensity or duration to the situation. Alongside these temper outbursts the child experiences a persistently irritable or angry mood most of the day, nearly every day. In order for diagnostic criteria to be met, the temper outbursts and irritable mood must be present for at least 12 months, across settings, and have an onset before the age of 10 years.

Early research into DMDD in the general population, where existing data have been used to derive diagnoses retrospectively, suggests that the prevalence ranges from 0.8% to 3.3% (Copeland et al., 2013). Children with DMDD have been found to be very impaired, with high rates of comorbidity including depression (Copeland et al., 2013, Dougherty et al., 2014). DMDD in the context of ADHD has not been studied widely. Results from community samples suggest that 4.3–23.5% of those with ADHD meet DMDD diagnostic criteria (Copeland et al., 2013, Mulraney et al., 2016). Therefore, the findings to date suggest that DMDD is more common in those with ADHD than in the general population. The only study that has examined the association between DMDD diagnosis and depression in an ADHD sample did not find any association with depression (Mulraney et al., 2016). However, this sample was pre-pubertal and so had not yet reached the age of risk for depression onset. Interestingly they did find an association with anxiety, an established pre-pubertal antecedent of depression (Pine and Fox, 2015).

If chronic childhood irritability is closely related to mood disorders, as conceptualised in DSM-5, then we would expect it to be associated with family history of depression and established pre-pubertal antecedents of depression in children with ADHD. Therefore, the aims of this study, which was based on a large clinical sample of children with ADHD, were to: 1) examine the prevalence of irritability, defined both as a continuous measure and categorically as DMDD, and 2) test associations between irritability and anxiety (symptoms or diagnosis), depression (symptoms or diagnosis), and family history of depression.

## 2 Methods

### 2.1 Sample

The sample consisted of 696 children aged 6–18 years (mean 10.9, s.d. 2.99) who took part in the Study of ADHD, Genes and Environment at Cardiff University, UK. This cross-sectional study recruited children with a clinical diagnosis of ADHD from UK child psychiatry and paediatric clinics between 2007 and 2011. All children also met DSM-IV or DSM-III-R research diagnostic criteria for ADHD. Children were all of British Caucasian origin and living with at least one biological parent at the time of the study. Exclusion criteria at study entry were any major comorbid neurological/neuropsychiatric disorder or genetic syndrome (including fragile X syndrome, tuberous sclerosis, epilepsy, psychosis, Tourette's syndrome and any known diagnosis of autism or other pervasive developmental disorder in keeping with DSM recommendations at that time). Children from these UK clinics scored across the IQ spectrum when tested during the study. The mean IQ was 83 (s.d=13.35), with 84 participants recording an IQ of <70. The majority of the sample (80.6%) was regularly taking stimulant medication at the time of the study. Ethical approval was obtained from the Wales Multicentre Research Ethics Committee. Written informed consent from parents and assent from children (or consent for those aged 16 years and older) were obtained for all individuals.

### 2.2 Measures

#### 2.2.1 Child psychopathology

The Child and Adolescent Psychiatric Assessment (CAPA) was completed with all parents. The CAPA is a semi-structured diagnostic interview that involves trained interviewers asking about symptoms of a wide range of psychiatric disorders present in the preceding 3 months (Angold and Costello, 2000). The symptoms asked about in the CAPA interview were designed to allow DSM-IV research diagnoses to be made based on the answers given. In order for a symptom to be endorsed on the CAPA it must be uncontrollable and interfere with at least 2 activities. All interviewers were trained to a high level of reliability (kappa=1.00 for agreement on ADHD diagnosis). All interviews were recorded and interviewers were supervised weekly by an experienced child and adolescent psychiatrist (AT).

The CAPA data, along with teacher reports (Child ADHD Teacher Telephone Interview (ChATTI) (Holmes et al., 2004), and Conner's Teacher Rating Scale (Conners, 1969)), were used to confirm the diagnosis of ADHD. The CAPA was used to ascertain other comorbid psychiatric disorders, and symptoms of these disorders, including depressive disorders (major depressive disorder and persistent depressive disorder (dysthymia)), common childhood anxiety disorders (generalised anxiety disorder and separation anxiety disorder), Conduct Disorder (CD) and Oppositional Defiant Disorder (ODD). Diagnoses were reviewed and updated according to DSM-5 criteria by two child and adolescent psychiatrists (OE and AT) in 2015.

Although the CAPA predates the addition of DMDD to DSM-5, the symptoms required for generating diagnoses are included in the interview and have been used previously to establish DMDD diagnosis (Copeland et al., 2013). As detailed in Table 1, if “losing temper” or “temper tantrum”, plus any one of “depressed mood”, “touchy or easily annoyed”, “angry or resentful” or “irritable” were present at the required frequency, duration and onset, the criteria for DMDD were met. For the diagnosis of DMDD, DSM-5 stipulates a number of exclusion criteria based on comorbidity. As a result, any participants who met diagnostic criteria for Bipolar Affective Disorder were excluded. However, as comorbidity was of interest in this study, other exclusions based on comorbidity were not applied.

CAPA reports were also used to derive a continuous measure of irritability by counting the presence or absence of the items “touchy or easily annoyed”, “angry or resentful” and “temper tantrums” (range: 0–3).

#### 2.2.2 Parent and family history of depression

To assess current parental depression the Hospital Anxiety and Depression Scale (HADS) (Zigmond and Snaith, 1983) was completed by mothers and fathers. Parents were recorded as having depression if they scored at or above the suggested cut point of 11 on the depression sub-scale of the HADS (Snaith, 2003).

Parents were also asked about any family history of depression. This included lifetime history of depression in the parent, as well as any history of depression in other first and second degree relatives of the child. A score of 1 was given for each first-degree relative (parent or sibling) and a score of 0.5 for each second-degree relative (grandparent, aunt or uncle, nephews or nieces or half siblings). The total provided a family history score for each child weighted by relatedness (Milne et al., 2009).

#### 2.2.3 Other measures

Demographic information (child gender, age and family income) was obtained through parent completed questionnaires. All children completed the Wechsler Intelligence Scale for Children (WISC-IV) (Wechsler, 2003), providing a full scale IQ for each child. The CAPA was used to measure impairment in the child. For each section of the CAPA, if a parent reported any symptoms as present in the child, they were asked if the symptoms interfered in different areas of their child's life (at home, in social interactions, in activities in the community, at school, in sports/clubs, in taking care of themselves, in play/leisure activities and in handling daily chores/responsibilities). If the symptoms interfered “sometimes” or “often”, then impairment was counted as present in that area of the child's life. The number of areas of the child's life where impairment was present (using information from all sections of the CAPA) was added up to make a total. This information allowed an overall child impairment score of 0–8 to be calculated.

### 2.3 Analyses

Data were analysed using SPSS version 20. Chi squared and independent samples t- tests were used to compare children with DMDD to those without DMDD on a number of demographic (child age, gender, IQ, family income), and clinical (ODD, CD, child impairment score) factors. Although ODD cannot coexist with DMDD according to DSM-5, its association with ODD was of interest in this study, so this exclusion was not applied. As the overlap between DMDD and ODD is predominantly due to irritable symptoms, the association between DMDD and *non- irritable* ODD symptoms was also examined. These non-irritable ODD symptoms have previously been identified as part of a headstrong/hurtful dimension of ODD (Stringaris and Goodman, 2009b). The prevalence of DSM-5 disorders in the sample was also examined according to child age and ADHD subtype as part of the supplementary analysis.

A series of univariate regression analyses were carried out to examine the association between irritability and depression related measures. Predictor variables were irritability score or DMDD diagnosis. Dependent variables were current child anxiety symptoms or diagnosis, depression symptoms or diagnosis, current mother or father depression, and family history of depression. These analyses were also run controlling for child age, family income and impairment. We also conducted a sensitivity analysis excluding participants with IQ <70 (see Supplementary information). As “depressed mood” and “irritable” CAPA items were used in the algorithm for DMDD diagnosis, there was a potential for associations between DMDD and depression related outcome measures to be inflated. Therefore, the regression analyses were also run with the “depressed mood” and “irritable” symptoms excluded from the DMDD algorithm. A sensitivity analysis was also carried out to test whether the headstrong/hurtful symptoms of ODD were associated with the depression related measures.

A number of variables were skewed (child impairment, family history of depression, child depression symptom and child anxiety symptom score variables) so analyses were conducted using both transformed and untransformed variables. As the results did not differ following transformation, the untransformed scores were reported for ease of interpretation.

## 3 Results

The mean age of the sample was 10.9 years (range 6–18 years, s.d 2.99), and 84% of the sample were male. Approximately 74% of children had a diagnosis of ADHD- combined type, 6% had a predominantly inattentive presentation, and 9% had a predominantly hyperactive-impulsive presentation (DSM-5 criteria). Eleven percent met diagnostic criteria for ADHD DSM-III-R (DSM-III-R diagnosis was used in the original study where teacher reports were unobtainable to confirm pervasiveness of ADHD symptoms). A total of 4.2% of children met diagnostic criteria for any depressive disorder (1.9% Major Depressive Disorder and 2.7% Persistent Depressive Disorder −0.4% had both), and 6.1% for any anxiety disorder in the 3 months prior to the assessment. Twenty one percent of mothers and 11% of fathers were above the cut off for current depression on the HADS.

Complete DMDD data were available for 95% of the sample (662 out of the 696). Of the 34 participants where complete data were not available, 18 had the required number of symptoms for DMDD diagnosis but information about pervasiveness across settings was missing. The other 16 did not have enough symptoms recorded for the presence or absence of DMDD to be established. Therefore, a final sample of 662 was used in the DMDD analyses. A total of 97% (678 out of 696) had information recorded for all 3 of the irritable symptoms, so these were included for the irritability score analyses.

Based on parent report, symptoms of irritability were common in this sample. The mean irritability symptom score was 2.19 (s.d 1.0), with 9% of the sample having reported no symptoms, 15% having one symptom, 23% having 2 symptoms and 53% having the maximum of 3 symptoms. In terms of the categorical DMDD diagnosis, the 3-month prevalence of the disorder was 31%. The numbers meeting each of the DMDD diagnostic criteria are reported in Table 2.

Children with a diagnosis of DMDD were significantly younger than those without (mean=9.9 *v.* 11.3 years, t(447)=6.04, p<0.001), and were more likely to have come from low income families (71% *v.* 59%, χ2=6.71, p=0.010). There was no difference between those with and without DMDD in child gender or IQ. Those with DMDD had a higher mean impairment score (mean=7.4 *v.*6.9, t(568)=4.91, p<0.001) and were more likely to have comorbid ODD (88.9% *v.* 33.5%, χ2=174.6, p<0.001) and CD (34.5% *v.* 11.0%, χ2=51.9, p<0.001) (Table 3). The association between DMDD and non-irritable symptoms of ODD (i.e. headstrong/hurtful dimension) was significant (unstandardized B=1.055, 95% CI=0.83, 1.28, p<0.001).

We explored the prevalence of DMDD and other comorbidities according to ADHD subtype and age. DMDD prevalence was 35.8% in those with ADHD combined type, 13.3% in those with a predominantly hyperactive-impulsive presentation and 10% in those with a predominantly inattentive presentation (see Supplementary material for more details). Rates of all comorbidities were highest in the ADHD combined presentation. The pattern of a higher prevalence in a younger age group was less marked for ODD than for DMDD.

Results from the regression analyses found that DMDD was associated with comorbid anxiety symptoms (unstandardized B=0.494, 95% CI=0.15, 0.84, p=0.006), anxiety disorder (OR=2.59, 95% CI=1.36, 4.93, p=0.04) and depression symptoms (unstandardized B=0.38, 95% CI=0.15, 0.60, p=0.001), but not depression diagnosis (OR=0.97, 95% CI=0.41, 2.26, p=0.940). The mothers of children with DMDD were more likely to be currently depressed than mothers of non-DMDD youth (27.1% *v.* 17.8%, OR=1.7, 95% CI=1.11, 2.65, p=0.016), but there was no difference in rates of current depression in the fathers (9.5% *v.* 9.6%, OR=0.99, 95% CI=0.41, 2.41, p=0.983). Children with DMDD had higher mean family history of depression score than those without (0.51 *v.* 0.40, unstandardized B=0.111, 95% CI=0.01, 0.22, p=0.04) (Table 4). As only 2 participants relied solely on the “depressed mood” or “irritable” symptoms of the CAPA in order to reach the threshold for DMDD diagnosis, all results remained the same when the “depressed mood” and “irritable” items were excluded from the DMDD algorithm.

The results were similar when irritability score was used as the predictor variable. Irritability score was also associated with child anxiety symptoms (unstandardized B=0.29, 95% CI=0.13, 0.44, p<0.001), anxiety disorder (OR=1.88, 95% CI=1.2, 2.96, p=0.006) and depression symptoms (unstandardized B=0.296, 95% CI=0.196, 0.395, p=<0.001). However, in addition, irritability score was associated with depression diagnosis (OR=2.8, 95% CI=1.36, 5.73, p=0.005). When the association was examined separately for Major Depressive Disorder (MDD) and Persistent Depressive Disorder, association was strongest for Persistent Depressive Disorder (OR=4.70, 95% CI=1.3, 17.0, p=0.018) and association with MDD alone did not reach conventional levels of statistical significance (OR=2.13, 95% CI=0.90, 5.04, p=0.086). Irritability score was also associated with family history of depression (unstandardized B=0.058, 95% CI=0.01, 0.11, p=0.021). Irritability score was not associated with current maternal (OR=1.12, 95% CI=0.9, 1.4, p=0.307) or paternal depression (OR=1.17, 95% CI=0.79, 1.73, p=0.444) (Table 5).

The overall pattern of results for the regression analyses remained similar when adjusting for age, low income and impairment, and when excluding those in the sample with an IQ<70. The only differences observed were that irritability (irritable score and DMDD) was no longer significantly associated with weighted family history of depression when adjusting for child age, family income and child impairment, and that DMDD was no longer significantly associated with current maternal depression when participants with low IQ were excluded (see Supplementary information).

When the headstrong/hurtful dimension of ODD was examined as a predictor variable instead of irritability, different results were found. The headstrong/hurtful dimension was not significantly associated with child anxiety symptoms or disorder, child depression diagnosis, or family history of depression. However, it was associated with child depression symptoms and current maternal and paternal depression (results available from first author).

## 4 Discussion

Our results suggest that symptoms of irritability and DMDD are common in children with ADHD, and that increased levels of irritability are associated with markers of depression liability.

Almost all children in the sample had at least one symptom of irritability, and the 3 month prevalence of DMDD diagnosis was 31%. This is considerably higher than in the general population (Copeland et al., 2013), and is also high when compared to the 4.3–23.5% prevalence in those with ADHD from community samples (Copeland et al., 2013, Mulraney et al., 2016).

When we examined the association between irritability and markers of depression liability, there were a number of significant findings. Associations were observed between irritability (both as a symptom score and DMDD diagnosis) and child depression symptoms, anxiety symptoms and anxiety disorder, as well as between irritable symptom score and depression diagnosis. These results support the hypothesis that irritability may be an early marker of mood problems in children with ADHD. Depression and anxiety symptoms in children have consistently been shown to be precursors for later depression.

Even though no association was found between DMDD and child depression diagnosis, this is not surprising given that depression is relatively rare in childhood. Risk for depression increases in adolescence (Thapar et al., 2012), but the mean age of our sample was 10.9 years. Therefore, our sample may have been too young to observe this association. These findings are consistent with those of Mulraney et al. (2016) who found that, in a community sample of children with ADHD aged 6–8 years, there was no association between DMDD and depression, but there was a significant association with anxiety. Despite this, we did find an association between irritability score and depression diagnosis. However, this seemed to be driven by the association with the Persistent Depressive Disorder (dysthymia) rather than Major Depressive Disorder (MDD). It is possible that as children get older and pass through the age of risk for depression the association between irritability and MDD will become stronger. Results showing associations between irritability and elevated risk of depression in the general population have mainly been based on longitudinal studies (Vidal-Ribas et al., 2016). Longitudinal studies following children with ADHD into adolescence and young adulthood are also needed if the relationship between irritability and depression in ADHD is to be examined.

Irritability score and DMDD diagnosis were also associated with other markers of depression liability. DMDD diagnosis was associated with a stronger family history of depression, and current maternal depression. Irritability score was associated with family history of depression. These findings provide further support for the hypothesis that irritability is related to mood disorders, specifically depression, in children with ADHD. However, irritability was not associated with all the family history variables measured. Irritability score in the child was not associated with current maternal depression, and neither irritability score nor DMDD diagnosis were associated with current paternal depression. Also, when the analyses were rerun adjusting for age, family income and child impairment the association with family history was no longer present. Previous studies in the general population also show mixed findings when looking at these associations. Some studies support the finding that child irritability is associated with maternal depression. For example, an irritable dimension of ODD was found to be associated with the presence of maternal depression in a community sample (Krieger et al., 2013). Also, children with more severe irritability trajectories have been shown to be more likely to have mothers with recurrent depression in a population-based cohort (Wiggins et al., 2014). However, other studies that examined the association between DMDD and parental psychopathology both in the general population and in clinical samples have not found any association (Dougherty et al., 2014, Axelson et al., 2012).

As DMDD in the context of ADHD has not been widely studied, we also examined a number of demographic and clinical characteristics of those with comorbid DMDD in this sample. We found that children with ADHD who also met diagnostic criteria for DMDD were significantly younger and were more likely to come from low income families. Results from previous studies have been mixed when looking at age and DMDD. In community samples some find higher rates in younger children (Copeland et al., 2013) while others find no difference (Mulraney et al., 2016). Lower rates of DMDD may be expected with increasing age, as prevalence of irritability decreases with age (Copeland et al., 2015). In terms of family income, our results are consistent with previous population studies which suggest that poverty is associated with DMDD (Copeland et al., 2013).

On examining clinical factors associated with DMDD, our findings suggest that children with ADHD and DMDD have higher rates of comorbidity than those without. These findings are in line with previous literature from community samples that suggest comorbidity is common in those with DMDD (Copeland et al., 2013, Dougherty et al., 2014). ODD was particularly common in our sample, with 89% of those meeting criteria for DMDD also meeting criteria for ODD. This compares to 90% in a community ADHD sample (Mulraney et al., 2016), and 96% in a clinical sample with elevated symptoms of mania (Axelson et al., 2012). High rates of comorbidity may be important to consider when examining the association between irritability and depression. This is because commonly comorbid conditions (e.g. ODD or anxiety disorders) are also known to be associated with depression. They could, therefore, have an impact on associations seen between irritability and depression. The effect of comorbidity was not examined in the current study, but irritability in the context of a depressive episode has been shown to predict a more severe, chronic and complex depressive illness which was not explained by comorbidity (Judd et al., 2013). Comorbidity will be an important factor to consider in future studies.

We also found that ADHD youth with DMDD are more impaired than those without. The mean impairment score was high across the whole sample, but higher in those with DMDD. Previous findings from a community ADHD sample have also found DMDD to be associated with high levels of impairment, particularly in social functioning (Mulraney et al., 2016). High levels of impairment have consistently been shown to be associated with irritability (e.g. Brotman et al., 2007; Shaw et al., 2014). Therefore, it is not surprising that DMDD is also associated with high levels of impairment in our sample. However, it is worth noting that, in an already impaired group of children with ADHD, having DMDD adds to the level of impairment they experience.

### 4.1 Limitations

There are a number of limitations to this study that should be noted. Firstly, this study used a large clinical sample of children with ADHD in order to address its aims. Although a clinical sample allowed a better understanding of DMDD and irritability within a population characteristic of those being treated for ADHD, there are biases within clinical samples, and our findings are not generalizable beyond this group. The study was also limited by the fact that the diagnosis of DMDD relied on a diagnostic interview that predated the introduction of DSM 5 and thus was not designed to make DMDD diagnosis. However, we used the same method as used for previous studies (Copeland et al., 2013), and the information available does allow the diagnostic criteria to be followed. It should also be noted that the “depressed mood” and “irritable” symptoms that were used to make DMDD diagnosis came from the depression section of the diagnostic interview. This could potentially increase the likelihood of association between DMDD and child depression symptoms or diagnosis. However, depression symptoms were relatively rare in this sample and only two participants relied on the presence of either the “depressed mood” or “irritable” symptoms to meet diagnostic criteria for DMDD. Therefore, when these symptoms were excluded from the DMDD diagnosis algorithm, there was no difference in any of the results. Another point to note when using DMDD diagnosis is that there has been some debate about its utility as a diagnostic category. It has been suggested that due to its overlap with other disorders and its lack of stability over time, more research is needed to establish whether it is a valid diagnostic category (Axelson et al., 2012, Ambrosini et al., 2013). It has a complex set of diagnostic exclusion criteria and it is not clear yet how helpful it will be in clinical practice. However, the aim of this study was not to evaluate the validity of DMDD as a diagnostic category, but to use it to understand more about a group of impaired children with ADHD and chronic irritability.

Another factor that is potentially important to take into account when examining the association between irritability and depression related factors in those with ADHD, is medication use. ADHD medication may have an effect on both symptoms of irritability and depression. For example, depression and emotional disturbance have been reported as possible adverse drug reactions in those taking ADHD medication (Aagaard and Hansen, 2011), whereas other studies have shown that irritability and depression symptoms improve in children with ADHD who take stimulant medication (Fernandez de la Cruz et al., 2015, Chang et al., 2016). As the vast majority of participants in our sample were taking regular stimulant medication the effect of medication could not be considered, but this is relevant for future work.

Another limitation is the fact that all the results reported in this study were based on parent only report. It is possible that if the parent reporting on their child's symptoms is depressed, it could bias findings. For example, a depressed mother may be more likely to report their child as irritable. Children's as well as parental reports of symptoms may have provided more information and reduced the risk of this bias. However the sample is relatively young and self-reports in those aged 11 and under can also be unreliable (Schwab-Stone et al., 1994). Finally, the study was also limited by the fact that parent psychopathology was assessed using questionnaire measures.

## 5 Conclusions

Overall, findings suggest that DMDD and irritability are common in children with ADHD, linked with substantial functional impairment affecting many aspects of children's lives, and may foreshadow future risk for depression as evidenced by associations with multiple known child and familial markers of depression liability (childhood depression and anxiety symptoms, maternal depression and family history of depression).

These findings have implications when seeing children with ADHD in clinical practice. They suggest that routine assessment of irritability in ADHD may be helpful for distinguishing those at highest risk for current impairment and future depression risk. Identifying these children may also be relevant when considering treatment. Although research is still limited, emerging evidence suggests that children with ADHD and irritable symptoms show improvement in their irritability when treated with stimulant medication (Fernandez de la Cruz et al., 2015). Preliminary work in children with chronic irritability and ADHD also suggests that a group therapy, incorporating components of cognitive-behavioural therapy with a parent-training intervention may be of benefit (Waxmonsky et al., 2013). Finally, the findings highlight the importance of longer-term monitoring of risk of developing depression as these children get older.

## Figures and Tables

**Table 1:** Defining DMDD using Child and Adolescent Psychiatric Assessment (CAPA).

**DMDD diagnostic criteria**	**CAPA items used**
Severe temper outbursts	Fulfilled if “losing temper” or “temper tantrum” items present in the ODD section.
Temper outbursts inconsistent with development	Fulfilled if either “losing temper” or “temper tantrum” items present in the ODD section.
Frequency of temper outbursts ≥3 x/week	Fulfilled if “losing temper” frequency total ≥36, or “temper tantrum” frequency total ≥36 (equivalent to the symptom being present on average at least 3x per week over the 3 month period that the CAPA asks about).
Irritable or angry mood (mood between outbursts is persistently irritable or angry)	Fulfilled if any of the following items from the depression section of the CAPA have a frequency of >45: “touchy or easily annoyed”, “angry or resentful”, “depressed mood” or “irritable” (equivalent to the symptom being present on more days than not over the 3 month period the CAPA asks about).
Temper outbursts and irritable mood present for >12 months	Fulfilled if “losing temper” OR “temper tantrum” present ≥3x/week for >12 months, AND “touchy or easily annoyed” OR “angry or resentful” OR “depressed mood” OR “irritable” symptom present on more days than not for >12 months.
Symptoms present in at least 2 settings	Fulfilled if “losing temper” or “temper tantrums” were present in at least 2 of the 3 settings asked about in the CAPA i.e. school, home or elsewhere.
Diagnosis not to be made before age 6 or after age 18 years	No children in this sample were <6years or >18 years.
Temper outbursts and irritable mood onset <10 years	Fulfilled if date of onset of required symptoms was before the child aged 10 yrs.

DMDD=Disruptive Mood Dysregulation Disorder; ODD=Oppositional Defiant Disorder.

Table 1 illustrates the method by which DMDD diagnosis was derived, based on DSM-5 diagnostic criteria and using the Child and Adolescent Psychiatric Assessment (CAPA). As comorbidity is of interest in this study, exclusions for DMDD diagnosis based on comorbidity weren't applied.

**Table 2:** Frequency of individual DMDD criteria and diagnosis.

**DMDD criteria**	**No. meeting criteria**	**(%)**
Severe temper outbursts (“losing temper” or “temper tantrums” present at CAPA interview)	630	92
Frequency of temper outbursts ≥3x/week	412	60
Irritable or angry mood present more days than not	388	57
Temper outbursts (≥3x/week) and irritable mood (present more days than not) for >12 months	282	42
Symptoms present in at least 2 settings	328	51
Temper outbursts (≥3x/week) and irritable mood (present more day than not) with an onset <10 years	258	38
**Full DMDD criteria met**	**207**	**31**

DMDD=Disruptive Mood Dysregulation Disorder; CAPA=Child and Adolescent Psychiatric Assessment

Table 2 shows the prevalence of DMDD (Disruptive Mood Dysregulation Disorder) symptoms and diagnosis in the study sample. Symptoms were common, but their prevalence decreased when the required frequency, duration and onset of symptoms was taken into account. 31% of the sample met criteria for DMDD.

**Table 3:** DMDD and associated demographic and clinical factors.

	**With DMDD (n≤207)**^a^	**Without DMDD (n≤455)**^b^	**Test statistic (df)**	**P value**
**Demographic factors**				
Gender (% male)	83.7	84.1	χ2=0.01	0.917
Age (mean, in years)	9.9	11.3	t=6.04 (447)	<0.001*
IQ (mean)	82	83.3	t=1.13 (602)	0.261
Income (% <£20,000/year)	71	59	χ2=6.71	0.010*
**Clinical factors**				
Comorbid ODD (%)	88.9	33.5	χ2=174.6	<0.001*
Comorbid CD (%)	34.5	11.0	χ2=51.9	<0.001*
Impairment score (mean)	7.4	6.9	t=4.91 (568)	<0.001*

DMDD=Disruptive Mood Dysregulation Disorder, ODD=Oppositional Defiant Disorder, CD=Conduct Disorder. df=degrees of freedom.

Table 3 compares those meeting criteria for DMDD to those without DMDD, on a number of demographic and clinical factors.

* P<0.05 was considered significant.

a For all variables n≥183 except income where n=166.

b For all variables n≥421 except income where n=372.

**Table 4:** Association between DMDD and child anxiety, depression and family history of depression.

	**With DMDD (n=207)**^a^	**Without DMDD (n=455)**^b^	**Test statistic (95% CI)**	**P value**
**Child Anxiety or Depression**				
Anxiety symptoms (mean)	1.23	0.78	B=0.494 (0.15, 0.84)	0.006*
Anxiety disorder (%)	10.5	4.3	OR=2.59 (1.36, 4.93)	0.040*
Depression symptoms (mean)	1.49	1.16	B= 0.38 (0.15, 0.60)	0.001*
Depressive disorder (%)	3.9	4.0	OR=0.97 (0.41, 2.26)	0.940
**Family History of Depression**				
Current maternal depression (% ≥11 on HADS)	27.1	17.8	OR=1.7 (1.11, 2.65)	0.016*
Current paternal depression (% ≥11 on HADS)	9.5	9.6	OR=0.99 (0.41, 2.41)	0.983
Weighted family history of depression (mean)	0.51	0.40	B=0.111 (0.01, 0.22)	0.040*

DMDD=Disruptive Mood Dysregulation Disorder. HADS=Hospital Anxiety and Depression Scale. CI=Confidence interval.

Table 4 examines the association between DMDD diagnosis, anxiety and depression in the child, and family history of depression. Child anxiety disorder includes generalised anxiety disorder and separation anxiety disorder. Depressive disorder includes Major Depressive Disorder and Persistent Depressive Disorder. Weighted family history of depression includes information about family history of depression in any first or second degree relatives of the child. A score of 1 was given for each first-degree relative and a score of 0.5 for each second-degree relative with the total providing a family history score weighted by relatedness.

Results show significant associations between DMDD and anxiety symptoms, anxiety disorder, depression symptoms, current maternal depression and weighted family history of depression. When analyses were rerun adjusting for age, family income and child impairment, all associations remained except the association with weighted family history of depression was no longer significant (unstandardized B=0.011, 95% CI=−0.02, 0.23, p=0.098) (see Supplementary information).

* P<0.05 was considered significant.

a For all variables n≥195 except current maternal depression where n=170 and current parental depression where n=84.

b For all variables n≥428 except current maternal depression where n=348 and current paternal depression where n=177. B represents the unstandardized B coefficient, OR represents the Odds Ratio.

**Table 5:** Association between irritability score and child anxiety, depression and family history of depression.

	**Test statistic (95% CI)**	**P value**
**Child Anxiety or Depression**		
Anxiety symptoms	B=0.29 (0.13, 0.44)	<0.001*
Anxiety disorder	OR=1.88 (1.2, 2.96)	0.006*
Depression symptoms	B= 0.296 (0.20, 0.40)	<0.001*
Depressive Disorder	OR=2.8 (1.36, 5.73)	0.005*
**Family History of Depression**		
Current maternal depression (≥11 on HADS)	OR=1.12 (0.9, 1.4)	0.307
Current paternal depression (≥11 on HADS)	OR=1.17 (0.79, 1.73)	0.444
Weighted family history of depression	B=0.058 (0.01, 0.11)	0.021*

HADS=Hospital Anxiety and Depression Scale. CI= Confidence interval. For all variables n≥635 except current maternal depression where n=530 and current parental depression where n=267. B represents the unstandardized B coefficient, OR represents the Odds Ratio.

Table 5 examines the association between irritable score, anxiety and depression in the child, and family history of depression. Child anxiety disorder includes generalised anxiety disorder and separation anxiety disorder. Depressive disorder includes Major Depressive Disorder and Persistent Depressive Disorder. Weighted family history of depression includes information about family history of depression in any first or second degree relatives of the child. A score of 1 was given for each first-degree relative and a score of 0.5 for each second-degree relative with the total providing a family history score weighted by relatedness.

Results show significant associations between irritable score and anxiety symptoms, anxiety disorder, depression symptoms, depressive disorder and weighted family history of depression. When analyses were rerun adjusting for age, family income and child impairment, all associations remained except the association with weighted family history of depression was no longer significant (unstandardized B=0.046, 95% CI=−0.01, 0.10, p=0.116) (see Supplementary information).

* P<0.05 was considered significant.
