# Supplementary material for: Irritability in ADHD: Associations with depression liability
Source: J Affect Disord. 2017 Jun;215:281–7. doi: 10.1016/j.jad.2017.03.050 (PMC5409953; doi:10.1016/j.jad.2017.03.050)
Supplement: Supplementary file 1 — Supplementary material [file mmc1.docx]

**Supplementary material**

1. **Sensitivity analyses testing associations between irritability (irritability score and DMDD) and depression related measures when excluding those with low IQ (<70).**

**Supplementary Table 1: Association between DMDD and depression related measures in full sample compared to sample excluding those with low IQ**

|  | **Full sample** | | | **Excluding all cases with IQ<70** | |
| --- | --- | --- | --- | --- | --- |
|  | **Test statistic**  **(95% CI)** | | **P value** | **Test statistic**  **(95% CI)** | **P value** |
| **Child Anxiety or Depression** | | | | | |
| Anxiety symptoms | B=0.494  (0.15, 0.84) | | **0.006*** | B=0.368  (-0.012, 0.75) | **0.006*** |
| Anxiety disorder | OR=2.59  (1.36, 4.93) | | **0.040*** | OR=2.24  (1.08, 4.65) | **0.031*** |
| Depression symptoms | B= 0.38  (0.15, 0.60) | | **0.001*** | B= 0.405  (0.16, 0.65) | **0.001*** |
| Depression diagnosis | OR=0.97 (0.41, 2.26) | | 0.940 | OR=1.22  (0.44, 3.36) | 0.699 |
| **Family History of Depression** | | | | | |
| Current maternal depression  (% ≥11 on HADS) | | OR=1.7  (1.11, 2.65) | **0.016*** | OR=1.33  (0.8, 2.2) | 0.276 |
| Current paternal depression (% ≥11 on HADS) | | OR=0.99  (0.41, 2.41) | 0.983 | OR= 1.3  (0.49, 3.48) | 0.595 |
| Weighted family history of depression | | B=0.111  (0.01, 0.22) | **0.040*** | B=0.158  (0.04, 0.28) | **0.009*** |

DMDD=Disruptive Mood Dysregulation Disorder. HADS=Hospital Anxiety and Depression Scale. CI=Confidence interval. B represents the unstandardized B coefficient, OR represents the Odds Ratio.

Table 1 compares the results of regression analyses in the full sample to results after excluding those with a low IQ (<70). Child anxiety disorder includes generalised anxiety disorder and separation anxiety disorder. Depression diagnosis includes Major Depressive Disorder and Persistent Depressive Disorder. Weighted family history of depression includes information about family history of depression in any first or second degree relatives of the child. A score of 1 was given for each first-degree relative and a score of 0.5 for each second-degree relative with the total providing a family history score weighted by relatedness.

*P<0.05 was considered significant.

The pattern of results remains similar when excluding participants with a low IQ. The only difference is that the association between DMDD and current maternal depression drops (OR 1.33 vs. 1.7) and no longer reaches statistical significance when participants with low IQ are excluded.

**Supplementary Table 2: Association between irritability score and depression related measures in full sample compared to sample excluding those with low IQ**

|  | **Full sample** | | **Excluding all cases with IQ<70** | |
| --- | --- | --- | --- | --- |
|  | **Test statistic**  **(95% CI)** | **P value** | **Test statistic**  **(95% CI)** | **P value** |
| **Child Anxiety or Depression** | | | | |
| Anxiety symptoms | B= 0.29  (0.13, 0.44) | **<0.001*** | B= 0.201  (0.33, 0.37) | **<0.033*** |
| Anxiety disorder | OR=1.88  (1.2, 2.96) | **0.006*** | OR=1.58  (1.01, 2.49) | **0.046*** |
| Depression symptoms | B= 0.296  (0.196, 0.395) | **<0.001*** | B= 0.292  (0.19, 0.40) | **<0.001*** |
| Depression diagnosis | OR=2.8 (1.36, 5.73) | **0.005*** | OR= 3.47  (1.24, 9.73) | **0.018*** |
| **Family History of Depression** | | | | |
| Current maternal depression  (% ≥11 on HADS) | OR=1.12  (0.9, 1.4) | 0.307 | OR= 0.99  (0.78, 1.26) | 0.946 |
| Current paternal depression  (% ≥11 on HADS) | OR=1.17  (0.79, 1.73) | 0.444 | OR=1.08  (0.71, 1.65) | 0.719 |
| Weighted family history of depression | B=0.058  (0.01, 0.11) | **0.021*** | B=0.070  (0.02, 0.12) | **0.010*** |

HADS=Hospital Anxiety and Depression Scale. CI=Confidence interval. B represents the unstandardized B coefficient, OR represents the Odds Ratio.

Table 2 compares the results of regression analyses in the full sample to results after excluding those with a low IQ (<70). Child anxiety disorder includes generalised anxiety disorder and separation anxiety disorder. Depression diagnosis includes Major Depressive Disorder and Persistent Depressive Disorder. Weighted family history of depression includes information about family history of depression in any first or second degree relatives of the child. A score of 1 was given for each first-degree relative and a score of 0.5 for each second-degree relative with the total providing a family history score weighted by relatedness.

*P<0.05 was considered significant.

The pattern of results remains similar when excluding participants with a low IQ.

1. **Sensitivity analyses testing associations between irritability (irritability score and DMDD) and depression related measures adjusting for age, income and impairment.**

**Supplementary Table 3: Association between DMDD and depression related measures adjusting for age, income and impairment**

|  | **Unadjusted** | | **Adjusting for age, income and impairment** | |
| --- | --- | --- | --- | --- |
|  | **Test statistic**  **(95% CI)** | **P value** | **Test statistic**  **(95% CI)** | **P value** |
| **Child Anxiety or Depression** | | | | |
| Anxiety symptoms | B=0.494  (0.15, 0.84) | **0.006*** | B= 0.544  (0.14, 0.95) | **0.008*** |
| Anxiety disorder | OR=2.59  (1.36, 4.93) | **0.040*** | OR= 2.52  (1.21, 5.26) | **0.014*** |
| Depression symptoms | B= 0.38  (0.15, 0.60) | **0.001*** | B=0.425  (0.17, 0.68) | **0.001*** |
| Depression diagnosis | OR=0.97 (0.41, 2.26) | 0.940 | OR= 0.97  (0.35, 2.66) | 0.950 |
| **Family History of Depression** | | | | |
| Current maternal depression  (% ≥11 on HADS) | OR=1.7  (1.11, 2.65) | **0.016*** | OR= 1.69  (1.04, 2.75) | **0.034*** |
| Current paternal depression (% ≥11 on HADS) | OR=0.99  (0.41, 2.41) | 0.983 | OR= 0.65  (0.22, 1.93) | 0.442 |
| Weighted family history of depression (mean) | B=0.11  (0.01, 0.22) | **0.040*** | B=0.11  (-0.02, 0.23) | 0.098 |

DMDD=Disruptive Mood Dysregulation Disorder. HADS=Hospital Anxiety and Depression Scale. CI=Confidence interval. B represents the unstandardized B coefficient, OR represents the Odds Ratio.

Table 3 compares the results of the unadjusted regression analyses to those adjusted for child age, family income and child impairment score. *P<0.05 was considered significant.

The pattern of results remains similar when adjusting for age, income and impairment. The only difference is that DMDD is no longer significantly associated with weighted family history of depression.

**Supplementary Table 4: Association between irritability score and depression related measures adjusting for age, income and impairment**

|  | **Full sample** | | **Adjusting for age, income and impairment** | |
| --- | --- | --- | --- | --- |
|  | **Test statistic**  **(95% CI)** | **P value** | **Test statistic**  **(95% CI)** | **P value** |
| **Child Anxiety or Depression** | | | | |
| Anxiety symptoms | B= 0.29  ( 0.13, 0.44) | **<0.001*** | B=0.25  (0.07, 0.43) | **0.006*** |
| Anxiety disorder | OR=1.88  (1.2, 2.96) | **0.006*** | OR=1.78  (1.08, 2.93) | **0.025*** |
| Depression symptoms | B= 0.296  (0.20, 0.40) | **<0.001*** | B=0.311  (0.20, 0.43) | **<0.001*** |
| Depression diagnosis | OR=2.8 (1.36, 5.73) | **0.005*** | OR=2.38  (1.17, 4.90) | **0.017*** |
| **Family History of Depression** | | | | |
| Current maternal depression  (% ≥11 on HADS) | OR=1.12  (0.9, 1.4) | 0.307 | OR=1.12  (0.88, 1.42) | 0.376 |
| Current paternal depression  (% ≥11 on HADS) | OR=1.17  (0.79, 1.73) | 0.444 | OR= 0.998  (0.64, 1.56) | 0.995 |
| Weighted family history of depression (mean) | B=0.058  (0.01, 0.11) | **0.021*** | B=0.046  (-0.01, 0.10) | 0.116 |

HADS=Hospital Anxiety and Depression Scale. CI=Confidence interval. B represents the unstandardized B coefficient, OR represents the Odds Ratio.

Table 4 compares the results of the unadjusted regression analyses to those adjusted for child age, family income and child impairment score. *P<0.05 was considered significant.

The pattern of results remains similar when adjusting for age, income and impairment. The only significant difference is that irritability score is no longer significantly associated with weighted family history of depression.

1. **Supplementary analyses examining comorbidity according to age group and ADHD subtype.**

**Supplementary Table 5: Comorbidities according to age and ADHD subtype**

|  | **Age ≤ 10 years**  **(n=338)** | **Age ≥ 11 years (n=358)** | **ADHD: combined (n=486)** | **ADHD: inattentive (n=41)** | **ADHD : hyperactive-impulsive (n=64)** |
| --- | --- | --- | --- | --- | --- |
| **Anxiety disorder** | 7.5% | 4.7% | 7.4% | 0% | 3.3% |
| **Depression diagnosis** | 2.8% | 5.5% | 4.5% | 2.6% | 4.8% |
| **ODD** | 55.1% | 49.3% | 57.8% | 5.0% | 32.8% |
| **CD** | 16.9% | 19.6% | 21.0% | 5.0% | 9.4% |
| **DMDD** | 39.4% | 23.7% | 35.8% | 10% | 13.3% |

ADHD=Attention-Deficit/Hyperactivity Disorder, ODD=Oppositional Defiant Disorder, CD=Conduct Disorder, DMDD=Disruptive Mood Dysregulation Disorder.
